# Health system changes needed to support people consulting general practice out of hours services in Ireland

**DOI:** 10.1186/s13033-018-0235-x

**Published:** 2018-10-13

**Authors:** C. Collins, M. T. O’Shea, J. Cunniffe, P. Finegan

**Affiliations:** 1 Irish College of General Practitioners, 4-5 Lincoln Place, Dublin 2, Ireland; 2 CareDoc, Carlow, Ireland

**Keywords:** Out of hours, Mental illness, General practice, Adherence, Follow-up

## Abstract

**Background:**

Mental illness acts as a barrier to accessing and obtaining effective medical care. It has been shown that out of hours services are an important first stop for emergency care for people experiencing mental health difficulties. However, little is in fact known about the use of out of hours general practice services by people experiencing mental health difficulties.

**Aim:**

To establish the number and range of consultations that have a primary or related mental health issue attending general practitioner (GP) out of hours and to document adherence to their follow-up care referral.

**Design and setting:**

Descriptive study in one large out of hours primary care service in the South East of Ireland (Caredoc).

**Methods:**

An anonymous extraction of retrospective data from 1 year of the out of hours’ electronic database was undertaken. Patients who attended the out of hours with a possible mental health issue and were referred to the psychiatric services or back to their own GP, were tracked via phone follow-up with hospitals and GPs over 6 months to establish if they attended for the recommend follow-up care.

**Results:**

Over a 1 year period, there were 3844 out of hours presentations with a mental health component. Overall, 9.3% were referred by the out of hours GP for follow-up to a hospital emergency department (ED) or were advised to attend their own GP. A total of 104 patients who were advised to attend their GP or ED following their consultation with the out of hours GP were tracked. Twenty-seven patients were referred back to their GP of which the follow-up call to the GP revealed that 44.5% did not attend. Seventy-seven patients were referred to the hospital services, of whom 37.7% did not attend.

**Conclusions:**

There are significant challenges at the interface of primary care and secondary mental health services in Ireland. As expounded by the WHO and WONCA, in order to be effective and efficient, care for mental health must be coordinated with services at different levels of care complemented by the broader health system.

## Background

Mental disorders are widespread with an estimated 25% of people experiencing such problems at some point in their lives and approximately 10% of the adult population experiencing a mental disorder at a given point in time [1]. Within mental health, depression affects approximately 5–10% of people and is the third most common reason for consultation in general practice [2]. By 2020, depression will be the second most common cause of disability worldwide [3].

Research suggests that morbidity and mortality rates are higher among individuals with serious mental health issues [4–11]. For those with a diagnosed mental illness, structural and systemic health disparities impact on access to and utilisation of health care [5]. Mental illness acts as a barrier to accessing and obtaining effective medical care [5].

There is unanimous consensus from the international literature that general practice has a central role in the provision of medical treatment and preventative health care to people with a severe mental illness [12–15]. The central role of primary care within the field of mental health is a global phenomenon with policy makers actively encouraging primary care to take a lead role in delivering mental health services [16]. In many countries, as in Ireland, the general practitioner (GP) is often the gatekeeper to secondary healthcare services whereby patients are obliged to see their GP first before being referred to specialised care. Identifying and providing the most suitable treatment for people with common mental health problems can be a difficult and complex process for health [17].

Much research into mental health has focused on psychiatry and emergency departments, with somewhat fewer concentrating on primary care. Within primary care, a further neglected service is that of out of hours. It has been shown that out of hours services are an important first stop for emergency care for people experiencing mental health difficulties [18, 19]. Out of hours service requirements for those experiencing mental health issues can also vary greatly from daytime service requirements, as patients more frequently present in crisis between the hours of 6 p.m. and 9 a.m. [19]. Little is in fact known about the use of out of hours GP services by people experiencing mental health difficulties. Out of hours GP services in Ireland operate from 6 p.m. to 9 a.m. Monday to Friday and on a 24 h basis over the weekend. Most of our hospitals and state mental health/psychiatric community services operate only during office hours. Hence, we consider that information on the patterns of use of out of hours GP services by people presenting with mental health difficulties and knowledge about their likely attendance following referral to mental health services will assist in informing the future development and structure of mental health services in Ireland.

There is a dearth of research on adherence to referrals by those presenting to GP out of hours services with mental health difficulties. A small number of studies focused on the general population’s adherence to follow-up care with their own GP [20, 21]. However, data is limited in terms of referral attendance. One Belgian study identified that in cases where suicide was attempted, individuals show limited compliance with referral for continuity of care [22].

This study outlines the frequency of consultations which had mental health as the primary or related reason for encounter at one large out of hours primary care service in the South East of Ireland (Caredoc) and presents data on whether patients attended for follow-up to the hospital or their own GP.

## Methods

The project consisted of two phases. In phase 1, data was collected via an anonymous extraction of retrospective data from the out of hours’ electronic database. All consultations which included any of the search terms in a pre-defined list of words (Table 1) that could be associated with a mental health issue in the notes of the call taker, the triage nurse or the attending GP were extracted to identify the number of patients over a 1 year period who attended with a possible mental health condition. The data extraction was undertaken by an employee of Caredoc following a pilot exercise related to 1 week of data in order to ensure inclusion of all search terms.

**Table 1:** Search terms

Depression	Suicide	Hallucinations
Depressed	Suicidal	Disturbed
Anxiety	Poison	Delirium
Depressive	Poisoning	Self harm
Bipolar	Psychosis	Self injury
Bi-polar	Psychotic	Agitated
Bi polar	Schizophrenia	Antidepressant
Mental	Schizophrenic	Anti-depressant
Psychiatry	Alzheimers	Anti depressant
Psychiatric	Dementia	

Phase 2 aimed to track patients who attended the out of hours with a possible mental health issue and following consultation with the out of hours GP needed referral to the psychiatric services via hospital emergency departments or back to their own GP for support in dealing with their mental health issue. It consisted of phone calls to hospitals and GPs over 6 months to establish if patients attended for advised follow-up care. Eligible patients were tracked using a specific flag created specifically for this purpose on the Caredoc electronic system. The out of hours doctor utilised this flag when a patient presenting was identified during the consultation with a possible mental health issue which required referral. Eligible patient lists were extracted and compiled by a pre-confirmed employee in Caredoc on a weekly basis over a 6 month period who then telephoned the ED departments and GPs to whom eligible patients were referred in the preceding week by Caredoc. The only information collected during this call was whether the individual attended the ED/GP following their Caredoc presentation. The study data did not subsequently contain any patient identifying information.

For the purpose of this study, the patient base was the GP out-of-hours in the South East, which consists of 390 member GPs and deals with over 280,000 episodes of care per annum in this area; with a population base of 550,000.

Descriptive analysis was undertaken using SPSS Version 22. A computerised search strategy was created to search the database for each term. Initially, a sample of 100 cases was also manually searched to check accuracy. Based on findings, all cases were subsequently manually reviewed.

## Results

**Fig. 1:**
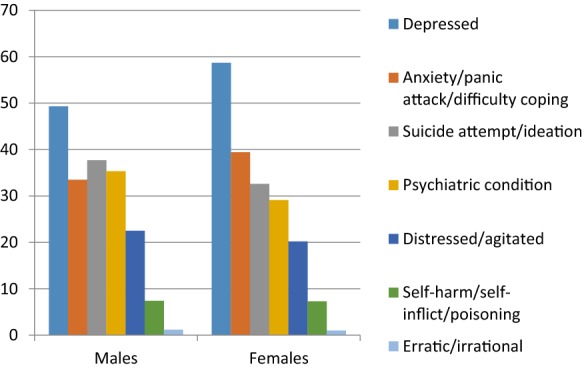
Prevalence of symptoms/diagnosis among males and females

In stage 1, using the pre-defined search terms outlined in Table 1, and excluding consultations relating to cases < 18 years (6.3%), 11,650 (8.6%) consultations containing a reference to a word listed in Table 1 took place in the out of hours service during the 1 year period, out of 135,103 total consultations with those aged 18 years and over. Overall 60.7% of these consultations related to females (Fig. 1); the mean adult age was 54.94 years (Table 2) and 48.6% of adult callers personally made the call to the service. The priority of the consultation at reception was considered an emergency for 8.5%, urgent for 36.8% and routine for the remaining 54.7% consultations.

**Table 2:** Age group—consultations

	n	%
18–24	1079	9.3
25–44	3452	29.6
45–64	2762	23.7
65–84	2883	24.7
85+	1474	12.7

The reason for encounter and/or the diagnosis is entered on the patient file as free text and not using a coding system in the out of hours service. The free text recorded by the call taker, the triage nurse and the attending GP was searched for specific terms. The inclusion and exclusion terms used to determine reasons for the consultation are shown in Table 3 resulting in 10,114 consultations.

**Table 3:** Included and excluded key word search and analysis terms

Included terms		
Depress (depression/depressed/depressive/antidepressant/anti-depressant/anti depressant) Abus (abusive/abused) Agitated Anxiety Anxious Breakdown Commit Cope Counsel Danger Delirious Delirium Distress	Disturb (disturb/disturbed/disturbing) Erratic Hallucination Involunt (involuntary) Kill Manic Mania Mood Mental (mentally) Overdos (overdose/overdosing/over dose) Paranoi (paranoia/paranoid) Panic Poison (poisoning)	Polar (bipolar/bi-polar/bi polar) Psychiatric Psychiatry Psychosis Psychotic Schizophren (schizophrenia/schizophrenic) Section (sectioned) Self harm Self inflict Self injury Suicid (suicide/suicidal) Voice (voices)

Excluded terms		
Mentally handicapped Mental handicap Mentally disabled Mental disability	Intellectually disabled Intellectual disability Not suicidal/nil suicidal No psychosis/nil psychosis	Not psychotic/nil psychotic Not depressed/nil depressed Alzheimers Dementia Mental scale

Multiple terms (relating to reason for encounter, diagnosis or symptoms) were possible to record within consultation notes—Table 4. Further exclusions and amalgamation of terms was undertaken; Table 5 outlines the resultant frequency of symptoms/diagnoses. Among these 3844 consultations, “depression” was noted in 54.7% of consultations, “anxiety” for 36.8%, risk of or threatening “suicide” for 34.8% and “psychiatric condition” in 31.7% of consultations.

**Table 4:** Occurrence of terms in consultations

	% of consultations
Depressed/depression/depressive/anti-depressant	51.8
Anxiety/anxious	30.4
Suicide/suicidal	29.8
Agitated	13.8
Psychiatry/psychiatric	11.6
Distressed	10.2
Mental	9.6
Low mood	8.8
Panic attack	8.5
Bi-polar	7.4
Self-harm	7.0
Psychotic/psychosis	6.9
Aggressive	6.4
Abuse	5.9
Kill	5.0
Schizophrenia/schizophrenic	4.5
Overdose	4.5
Paranoia	4.3
Difficulty coping	4.2
Counselling	4.1
Hearing voices	3.9
Commit	3.3
Involuntary admission	2.2
Breakdown	2.1
Violent	2.1
Danger	2.0
Disturbed	1.9
Hallucinations	1.7
Manic	1.6
Sectioned	1.5
Poison/poisoning	1.2
Delusional	1.1
Erratic behaviour	0.6
Irrational	0.4
Self inflict	0.3
Delirious	0.2

**Table 5:** Occurrence of symptoms/diagnoses in consultations

	% of consultations
Depression	54.7
Anxiety/panic/difficulty coping	36.8
Suicide attempt/ideation	34.8
Psychiatric condition^a^	31.7
Distressed/agitated	21.2
Self harm/self inflict	7.3
Erratic/irrational	1.1

^a^Bi-polar, psychosis, schizophrenia, delirious, sectioned, manic, delusional, having hallucinations, hearing voices, involuntary admission, paranoia, psychiatry, breakdown

**Fig. 2:**
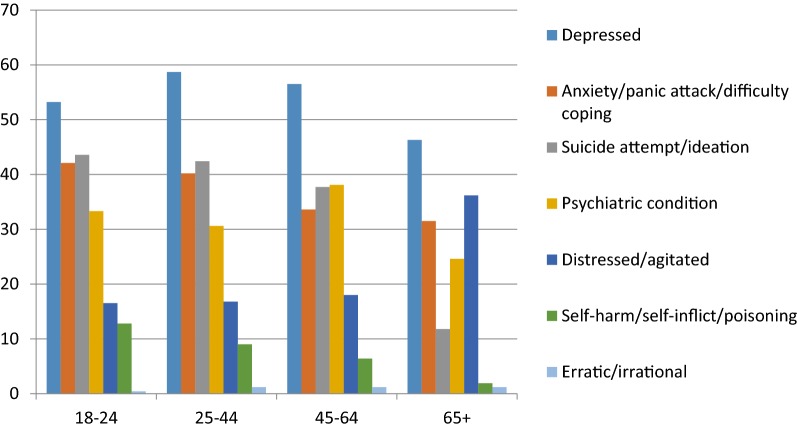
Prevalence of symptoms/diagnosis by age group

In terms of age groups, Fig. 2 shows prevalence in each age group with depression highest among those aged 25–44 years, anxiety and self-harm highest in those aged 18–24 years, suicide attempt/ideation marginally higher among those aged 18–25, followed by those aged 25–44 years, psychiatric conditions highest in those aged 45–64 years and prevalence of being distressed/agitated highest for the 65+ years old age group.

When data on referral was considered in the 3844 presentations, 356 patients (9.3%) were referred by the out of hours doctor for follow-up to a hospital emergency department or were advised to attend their GP. Those attending the out of hours with suicide attempt/ideation, self- harm or erratic/irrational behaviour were more likely than other groups to be referred for follow-up.

Over 6 months in the second phase of this project, a total of 104 patients who were recorded as having a mental health component to their consultation and advised to attend their GP or ED following their consultation with the out of hours GP were tracked. Twenty-seven patients were referred back to their GP of which the follow-up call to the GP revealed that 44.5% did not attend. Seventy-seven patients were referred to the hospital services, of whom 37.7% did not attend (Table 6).

**Table 6:** Phase 2—attendance and non-attendance to recommended follow-up

	Referred	Attended	Did not attend
Referred back to GP	27	15 (55.5%)	12 (44.5%)
Hospital A	28	20 (71.4%)	8 (28.6%)
Hospital B	31	17 (57.8%)	14 (45.2%)
Hospital C	18	11 (61.1%)	7 (38.9%)

## Discussion

### Summary

The findings from the first phase of this project sheds light on the number of patients presenting to an out of hours service with a complaint that has a mental health component. Searching the Caredoc database using 28 words that could indicate a mental health issue (excluding dementia and alzheimers), a total of 10,114 presentations were identified. Refining this further to include only those with clearly identifiable mental health symptoms/diagnosis resulted in 3844 consultations over the 1 year period. Phase 2, followed up on similar referrals over a 6 month period and showed that a substantially high number of patients who consult the out of hours service do not attend for advised follow-up care.

### Strengths and limitations

The main strength of this project is the provision of a picture of mental health consultation in GP out of hours, not heretofore available. While the numbers of cases referred for follow-up in phase 2 is below that estimated in phase 1, there are logical reasons for this. Phase 1 figures are based on key word searches whereas in phase 2 the doctor was recording referral status and this may have resulted in more stringent assignment. Furthermore, the Caredoc individual reviewing the cases in phase 2 and calling the EDs and GPs reassigned some of the cases tagged as referred by the consulting doctor due to vagueness in the notes, further potentially reducing the numbers noted as referred. A strength of the methodology was the depth of review of the database thereby ensuring more accurate enumeration.

### Comparison with existing literature

Despite the high prevalence of mental health issues presenting during consultations within primary care, a key barrier to detection and management is poor access to referral pathways [23, 24]. An important aspect of many care models is the linkage between primary and secondary care [25, 26]. Barriers impacting on effective collaboration between primary care and specialist services in relation to detection and treatment of depression occur at three levels: provider level, system level and patient level [27, 28]. Among provider level barriers, are the lack of adequate training as identified in previous research in Ireland along with deficiencies in protocols for the delivery of mental health care in the community [29]. Within the system level barriers, the absence of or limited access to a range of referral pathways for those not requiring specialist services are indicated [28]. Patient level barriers included the reluctance of individuals to engage with specialist mental health services due to associated stigma, alternative health beliefs and those who prefer to continue treatment with their primary care physician [28]. This may explain in part why two out of every five patients overall in our findings did not attend for the recommended follow-up.

The WHO and WONCA recommend an integrated approach to primary mental health care as the most viable way of closing the treatment gap [30]. This involves breaking down the interface boundaries and goes beyond collaboration and good communication across the primary–secondary care interface, to coordination and co-location of care [31]. However, it is recognised that information on the prevalence of mental health problems in primary care and the range of interventions provided in primary care is needed to effectively plan primary care services and the interface between primary care and specialist mental health services [32].

### Implications for research/practice

Coding of the reason for encounter and diagnoses within the out of hours system would improve the collation of data. Additional components including longitudinal data on the outcomes of those who attended and those who did not attend the recommended follow up appointment with their own GP or hospital ED and research on those who self-refer to hospital ED would add to the evidence base further.

The absence of intermediary alternatives to specialist/secondary level services within the Irish context results in referral and over reliance on more expensive specialist services. When considering attendances to out of hours services, low compliance with referral and follow-up must be taken into account when planning service provision [20–22].

## Conclusions

There are significant challenges at the interface of primary care and secondary care [33] and is so for mental health services in Ireland [32]. As expounded by the WHO and WONCA, in order to be effective and efficient, care for mental health must be coordinated with services at different levels of care complemented by the broader health system [30]. Concrete examples of how this could be achieved are through the establishment of a single point of contact with local mental health services for GPs in each area [32], dedicated mental health staff working within a consultant-led multidisciplinary liaison psychiatry team providing support to out-of-hours service providers [34] and the availability of mental health professionals in the primary care setting [32].
